# The effectiveness of decision-making support methods during pregnancy regarding epidural analgesia in labor: A scoping review

**DOI:** 10.18332/ejm/204274

**Published:** 2025-05-30

**Authors:** Yaya Kishimoto, Kayo Ueda, Toshiko Igarashi, Yuu Tanaka, Masahiko Kawaguchi

**Affiliations:** 1 Department of Nursing Women’s Health and Midwifery, Faculty of Nursing, Nara Medical University School of Medicine, Kashihara, Japan; 2 Department of Anesthesiology, Heisei Mahoroba Hospital, Kashihara, Japan; 3 Department of Anesthesiology, Nara Medical University, Kashihara, Japan

**Keywords:** analgesia, epidural, decision-making, pregnancy

## Abstract

**INTRODUCTION:**

This scoping review aimed to identify effective methods used for decision-making support and their effects on women considering choosing epidural analgesia in labor.

**METHODS:**

A literature search was conducted using CHNAHL, the Web version of the *Central Medical Journal*, and PubMed, and articles were extracted based on the eligibility criteria in September 2024. The PRISMA-ScR was followed. Studies that compared two groups of women considering epidural anesthesia for labor (subject), decision-making support (intervention), and standard care (control) were eligible.

**RESULTS:**

The search identified a total of 732 articles, of which 15 were eligible. Among these, 10 were randomized controlled trials (RCTs), 1 was a non-RCT, and 4 were cohort studies. The most common decision support approach was the distribution of leaflets, which were often given in the second to third trimester of pregnancy. Outcome measurements varied, limiting the identification of the most effective approach. However, most interventions, including controls, showed some effect on improving satisfaction and knowledge. Specifically, interventions incorporating shared decision-making (SDM) influenced satisfaction with the intervention, satisfaction with the birth experience, the feeling of being supported by others, and controlling emotions and attitudes.

**CONCLUSIONS:**

This study found that decision support methods include both expert intervention and media-based approaches. However, given the scarcity of related studies and the variability in evaluation measures, the most effective method could not be identified. Nevertheless, an SDM approach may enhance effectiveness. These findings may help those who support pregnancy and childbirth in choosing more effective decision support methods.

## INTRODUCTION

Despite the existence of individual variations, labor pains are experienced during delivery. Recently, epidural anesthesia has been listed as an option for childbirth to relieve labor pains^1^. Studies on women’s expectations of pain relief reported that women wanted to access effective pain relief, and a wide range of preferences was identified, ranging from women wanting no drugs at all during labor to those requesting sufficient drugs to make it a manageable or pain-free experience^2^. The new World Health Organization^3^ (WHO) 2020 childbirth care guide also recommends epidural analgesia for healthy pregnant women requesting pain relief during labor depending on a woman’s preferences^3^. A survey of medical facilities indicated the number of births performed with epidural anesthesia in Japan in 2020 was approximately 8.6% of the total 69933 cases^4^. In 2023, it accounted for 13.8% of the total number of 59026 cases^4^ and is continuously increasing. Using epidural anesthesia to relieve labor pains is often an elective medical procedure; therefore, the actual number of anesthesia-assisted births in Japan is estimated to be even higher.

Decision aids are also sometimes referred to as decision support tools. The international standard for decision support tools is called the International Patient Decision Aid Standards. Decision aids are used for making complex decisions that require extensive information and thorough deliberation. Complex decisions often present various options with features that people value differently. Sometimes the scientific evidence regarding these options is insufficient. Therefore, the best choice depends on the personal importance that the patient places on the benefits, harms, and uncertainties of scientific data^5^. Previous studies have used a combination of intentional and unintentional decision aids to help women plan their birth^6^. Intentional decision aids are believed to be necessary for women considering the use of epidural anesthesia.

Women considering the use of epidural anesthesia during delivery need to make their own choices with correct knowledge in addition to their values and background^7^. Support from medical professionals is important to obtain accurate knowledge. The suggested formats for epidural information were pamphlets or handouts, a list of trustworthy websites, question-and-answer leaflets, Internet-based information, other women’s experiences, and a video demonstrating epidural catheter placement^8^. However, no literature has clarified their effectiveness or the content and timing of effective interventions. Consequently, this study will systematically gather relevant literature and conduct a scoping review to review existing knowledge on decision support and its effectiveness while exploring effective support strategies in clinical settings. We believe this study will help clinicians and educators select the multiple decision support methods that are most appropriate for their practice.

Our research objective was to determine the current methods of decision aids and their impact on women considering epidural analgesia during delivery and to identify areas requiring further research in midwifery care for women opting for epidural analgesia.

## METHODS

### Study design and literature search

This scoping review followed the Preferred Reporting Items for Systematic Reviews and Meta-Analyses Extension for Scoping Reviews^9^.

A protocol was created and registered with the University hospital Medical Information Network (UMIN) Center on 9 September 2024 (UMIN Study ID: UMIN000055460). A literature search was conducted using CHNAHL, the web version of the *Central Medical Journal* on 13 September of the same year, and PubMed on 17 September of the same year, and data extraction was completed in September. A health science information specialist with experience in literature searches was provided with guidance for the search. The eligibility criteria were as follows: studies that employed decision support interventions targeting pregnant women considering epidural analgesia, regardless of their pregnancy stage or whether spontaneous or induced labor was occurring, in comparison with standard care or not employing decision-making interventions. Reviews were excluded.

With regard to definitions, epidural birth was noted as ‘Vaginal birth with epidural anesthesia’ while decision support was noted as ‘To support the right of women/families to actively participate in decisions about their care^10^, this study specifically addresses support for deciding whether to receive epidural analgesia’.

### Data extraction, synthesis, and presentation

This study was prepared following the reporting guidelines for scoping reviews (PRISMA-ScR)^9^ using a population, concept, context approach: the ‘population’ was ‘low-risk pregnant women considering their mode of delivery’, the ‘concept’ was ‘what is known about decision aids during pregnancy and their effectiveness’, and the ‘context’ consists of ‘hospitals and clinics that administer anesthesia during pregnancy and childbirth’. The search terms set for each database included the following keywords: ‘anesthesia’, ‘decision-making’, and ‘pregnant women’ (Supplementary file Table 1). The literature in English was comprehensively reviewed. Based on the article eligibility criteria, two authors screened the titles, abstracts, and full texts and then selected eligible articles for analysis. The authors settled differences in opinions through discussion and then decided the target articles.

The extracted data were: authors, publication year, country, sample characteristics, timing of the intervention, intervener, content of the interventions for the intervention and control groups, outcome measures, and presence of any significant differences. The analysis was based on the following research questions: 1) ‘What methods are used to support decision-making?’; 2) ‘When is the most effective time to provide decision support?’; 3) ‘What indicators are used to measure the effectiveness of decision support?’; 4) ‘Who are the effective implementers of decision support?’; and 5) ‘What are the effective methods for decision support?’.

## RESULTS

**Figure 1:**
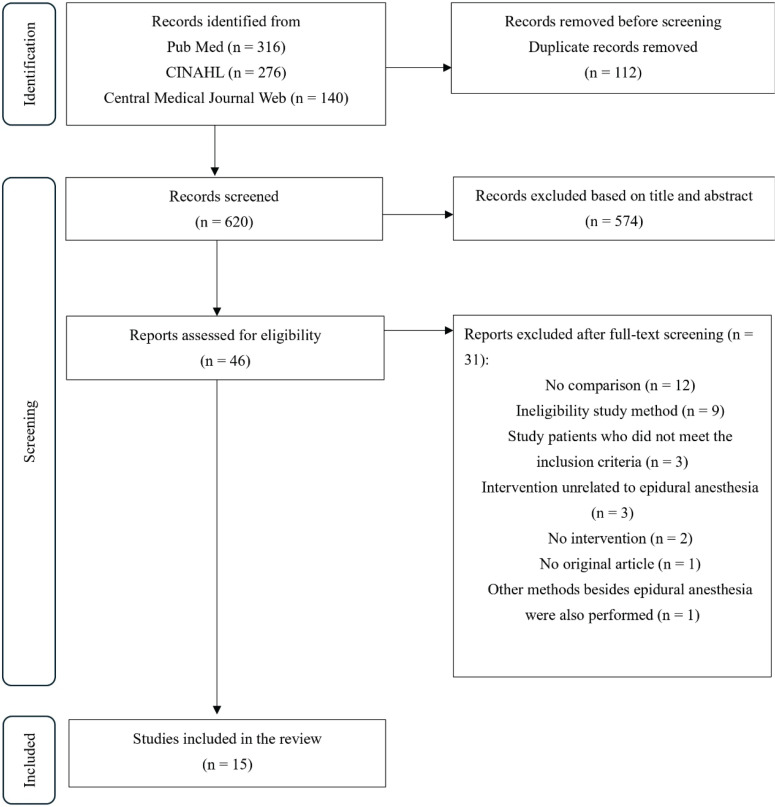
PRISMA flow diagram showing selection of included studies

A total of 732 articles that met the search criteria were included. After removing 112 duplicates, 15 articles^11-25^ were finally selected based on the inclusion criteria (Figure 1). Of the 15 studies, 10 were RCTs^11,13,14,18,20-25^, 1 was a non-RCT^15^, and 4 were cohort studies^12,16,17,19^. The total number of participants was 7258, and studies published between 1994 and 2024 were included. The included studies were conducted in Japan (1)^15^, China (1)^16^, Iran (1)^14^, Australia (3)^19,21,22^, Canada (1)^11^, United States (1)^23^, France (1)^12^, Denmark (2)^13,20^, United Kingdom (1)^18^, Spain (2)^24,25^, and Sweden (1)^17^ (Table 1).

**Table 1:** Characteristics of final selected literature on effectiveness of decision-making support during pregnancy regarding epidural analgesia labor

*Authors Year Country*	*Study design*	*Sample characteristics*	*Intervention*			*Control intervention*
			*Implementer*	*Timing*	*Details*	
Munro et al.^11^ 2018 Canada	RCT	Intervention group: Not clear Control group: Not clear Total: 40 English-speaking women who were pregnant or had given birth in the past 12 months	Not clear	During pregnancy or after childbirth	A short version of the pamphlet (846 words)	A long, detailed version of the pamphlet (1565 words)
Cherel et al.^12^ 2022 France	Cohort	Intervention group: 188 Control group: 178 Total: 366 All parturient women aged >18 years	Anesthetist nurse, anesthesiologist	Before birth (just before the anesthesia consultation)	Digital presentation Discussion Presentation of the equipment used by the anesthetist for epidural catheter placement and by parturient women for patient-controlled epidural analgesia	Conventional information provided by anesthesiologists
Maimburg et al.^13^ 2010 Denmark	RCT	Intervention group: 587 Control group: 575 Total: 1162 Nulliparous women aged >18 years at enrollment with singleton pregnancy, and the ability to speak and understand Danish	Midwives	Between 30 and 35 weeks of gestation	Lectures Discussion	Lessons on the birth process
Shahveisi et al.^14^ 2023 Iran	RCT	Intervention group: 33 Control group: 33 Total: 66people Women with a low-risk pregnancy and symptoms of labor	Midwives	Gestational age of 38–42 weeks	Individual counseling based on SDM Explanation of the advantages and disadvantages of using pharmacological and non-pharmacological pain relief methods	Routine delivery care
Shishido et al.^15^ 2020 Japan	Non-RCT	Intervention group: 149 Control group: 150 Total: 299 Low-risk women of singleton pregnancies who were planning to have vaginal delivery were between 34 and 36 gestation weeks before visiting the obstetric anesthesiology unit and could communicate, read, and write in Japanese	Not clear	After 34 weeks of pregnancy	The DA consists of 22 pages of A4-sized paper with: 1) Information on epidural anesthesia or no epidural anesthesia options, 2) a comparative table of each option, 3) a value clarification exercise, and 4) a decision-making process	Standard information pamphlet (the benefits and risks of epidural anesthesia)
Cheng et al.^16^ 2020 China	Cohort	Intervention group: 113 Control group: 111 Total: 224 Parturients aged ≥20 years had used epidural anesthesia during the natural birth process and could read Chinese or communicate in Mandarin or Taiwanese	Not clear	28th week of gestation	To provide the SDM parturient health education as well as a leaflet with quick response codes Scanning the code would link to an educational videocomp that explained what epidural analgesia is and its advantages and disadvantages	Explanation of analgesia after admission for delivery
Fabian et al.^17^ 2005 Sweden	Cohort	Intervention group: 1096 Control group: 101 Total: 1197 Swedish-speaking pregnant women	Midwives	After an early pregnancy	Childbirth and parent education classes (antenatal classes)	Those who did not attend antenatal classes
Stewart et al.^18^ 2003 England	RCT	Intervention group: Not clear Control group: Not clear Total: 76 Primiparous women	Not clear	12–14 weeks of gestation	Providing standard booking information and the OAA leaflet	Providing standard booking information
Swan et al.^19^ 1994 Australia	Cohort	Intervention group: 16 Control group: 24 Total: 40 Forty primipara women, none of whom had previously had an epidural	Anesthesiologist	Before epidural catheter insertion during labor	Antenatal epidural information (group education)	No antenatal epidural information
Brixval et al.^20^ 2016 Denmark	RCT	Intervention group: 883 Control group: 883 Total: 1766 Women aged ≥18 years, singleton pregnancy, and having the ability to speak and understand Danish	Midwives	25th, 33rd, and 35th weeks of pregnancy	Prenatal education in small classes, 6–8 people, including discussion among participants	A standard education with up to 250 participants
Raynes-Greenow et al.^21^ 2010 Australia	RCT	Intervention group: 395 Control group: 201 Total: 596 Primiparous women, in their final trimester, who were planning a vaginal birth of a single infant	Not clear	Late pregnancy	Booklet Audio guide (audio guidance to help with decision-making)	Booklet only
Raynes-Greenow et al.^22^ 2009 Australia	RCT	Intervention group: 193 Control group: 202 Total: 395 Women having their first baby (primiparous), late in pregnancy (≥36 weeks of gestation), who were planning for a vaginal birth of a single infant	Not clear	Around 36 weeks of pregnancy	Booklet CD	Booklet only
Togioka et al.^23^ 2019 USA	RCT	Intervention group: 50 Hispanic and 50 non-Hispanic Control group: 50 Hispanic and 50 non-Hispanic Total: 200 The patients were English- or Spanish-speaking Medicaid beneficiaries at least 18 years of age with a singleton fetus of at least 24 weeks of gestation and were non-midwife parturients presenting in spontaneous labor, having an induction of labor, or receiving augmentation of labor who were free to choose epidural labor analgesia	Resident doctors, nurses, and midwives	After 24 weeks of pregnancy	Video show Corresponding pamphlet Face-to-face counseling	Routine care (including counseling)
López-Gimeno et al.^24^ 2022 Spain	RCT	Intervention group: 227 Control group: 188 Total: 415 Women aged >18 years with low-medium obstetric risk underwent prenatal and postpartum controls in one of the participating Primary Care Units and underwent childbirth in an NHC reference hospital	Midwives	24–40 weeks pregnant	A midwife (who was trained in SDM) counseling the pregnant women about the elaboration of birth plans based on SDM An information leaflet	Standard counseling by midwives about the birth plan
López-Gimeno et al.^25^ 2024 Spain	RCT	Intervention group: 193 Control group: 223 Total: 416 Pregnant women with low-to-medium obstetric risk	Midwives	29–33 weeks pregnant	A midwife (who was trained in SDM) counseling the pregnant women about the elaboration of birth plans based on SDM A handout	Standard counseling by midwives about the birth plan

RCT: randomized controlled trial.

### What methods are used to support decision-making?

Of the 15 studies, leaflets were the most common intervention used (nine studies)^11,15,16,18,21-25^ followed by counseling (four studies)^14,23-25^, discussion (three studies)^12,13,20^, lectures^13,14^, video^16,23^, group education^17,19^, audio guide^21,22^ (two studies each) and presentation^12^. Four of the 15 studies clearly indicated that the decision support used a method based on shared decision-making (SDM)^14,16,24,25^.

### When is the most effective time to provide decision support?

The timing of intervention was from early pregnancy, mid-pregnancy, and late pregnancy onward in two^17,18^, three^20,23,24^, and nine studies^12-16,19,21,22,25^, respectively, while the rest were conducted before birth; however, the timing was unclear^11^ (one of these studies was literature questioning knowledge and included postpartum participants^11^). The timing of intervention was more frequently performed in mid or late pregnancy than in the early stages. No studies compared the effects of different intervention timings.

### What indicators are used to measure the effectiveness of decision support?

The following eight items were used: satisfaction with the intervention^12,15,16,18,21,22,24^, satisfaction with the birth experience^12-14,16,17,24^, knowledge and misunderstandings^11,12,15,19,22,23^, anxiety and fear^13,21,22^, conflict^15,21,22^, degree of participation in the decision-making process^21,22^, feeling supported by others, control of emotions and attitudes^14^, and planned to or decided to use epidural analgesia during delivery^11,13,15,17,20,21,23-25^.

### Who are the effective implementers of decision support?

Regarding the personnel who implemented the intervention, of the 15 studies, the interventions were carried out by midwives alone in six studies^13,14,17,20,24,25^, a doctor alone in one^19^, a medical team consisting of nurses or midwives and a doctor in two^12,23^, and a leaflet or other media alone in six^11,15,16,18,21,22^ without involvement of any professionals. No studies have compared the effects of interventions between professions (Table 1).

### What are the effective methods for decision support?

*Satisfaction with the intervention*

A total of seven studies^12,15,16,18,21,22,24^ measured satisfaction, and the interventions implemented were a combination of presentation and discussion^12^, a leaflet only^15,18,24^, a combination of leaflet and video^16^, a combination of leaflet and audio guide^21^, and a combination of leaflet, video and counseling^24^. Of these, four studies^12,15,16,21^ reported a significant difference in effectiveness, and the interventions implemented in effective studies were a combination of presentation and discussion^12^, a leaflet only^15^, a combination of leaflet and video^16^, and a combination of leaflet and audio guide^21^. Even in three studies without significant differences, the proportion of people who felt satisfied was higher than in the control group^18,22,24^. Media-only interventions were not associated with the decrease in satisfaction^15,16,21^, and compared with providing only information, a combination of a leaflet and video incorporating SDM techniques was associated with higher satisfaction with the intervention^16^ (Table 2).

**Table 2:** Scale used to measure tool and outcomes of effectiveness of decision-making support during pregnancy regarding epidural analgesia labor

*Authors Year Country Study design*	*Scale*	*Significant difference*	*No significant difference*
**Satisfaction with the intervention**			
Cherel et al.^12^ 2022 France Cohort	‘Satisfaction with the information regarding epidural analgesia received at the hospital during their pregnancy’ was assessed on a 10-level numeric scale	The intervention group had higher satisfaction levels (p<0.001)	
	‘Satisfaction with the efficacy of epidural analgesia’ was assessed on a 10-level scale		Same for both groups (p=0.21)
Shishido et al.^15^ 2020 Japan Non-RCT	DCS ^a^ subscale	A significant difference in satisfaction with the decision-making score was observed between the two groups (p<0.001)	
Cheng et al.^16^ 2020 China Cohort	English questionnaires adapted questions from PreMaPEQ ^b^, P-BESS ^c^, and WOMBLSQ ^d^	The scoring of questions related to satisfaction with the information provided were all higher in the intervention group (p<0.05)	
Stewart et al.^18^ 2003 Engrand RCT	To ask to score from 1 to 5 on the sources of information they had found most useful		30 (81%) women in the intervention group and 25 (64%) in the control group thought they had received adequate information (p=0.16)
Raynes-Greenow et al.^21^ 2010 Australia RCT	SWD ^e^ To ask whether women would recommend the materials ascertained	This was significantly higher among women in the intervention group (89.2%) compared with the control group (79.8%) (RR=1.34; 95% CI: 1.06–1.69)	Both groups would recommend the intervention they received to a pregnant friend (p=0.57)
Raynes-Greenow et al.^22^ 2009 Australia RCT	DCS ^a^ subscale		Women in the intervention group were slightly more likely to feel ‘satisfied with their decision-making’ (83%) compared with the control group (80%); no significant difference was observed between the two groups (p=0.112)
López-Gimeno^24^ et al. 2022 Spain RCT	MCSRS ^f^		Sufficient information on childbirth was received during pregnancy according to 95.1% (n=212) of the women in the intervention and 94.8% (n=183) in the control group
**Satisfaction with the birth experience**			
Cherel et al.^12^ 2022 France Cohort	‘Global satisfaction with their delivery’ was assessed on a 10-level numeric scale	Increased in the intervention group during the second period (p=0.01)	
Maimburg et al.^13^ 2010 Denmark RCT	The women’s own birth experience (measured on a five-point Likert scale)		No significant differences in birth experience
Shahveisi et al.^14^ 2023 Iran RCT	LAS ^g^, MCSRS ^f^	The mean score of childbirth satisfaction in the intervention group was significantly higher than that in the control group (p<0.001)	
Cheng et al.^16^ 2020 China Cohort	English questionnaires adapted questions from PreMaPEQ ^b^, P-BESS ^c^, and WOMBLSQ ^d^	Some questions indicated higher satisfaction scores for the SDM group about the service in the labor room and the delivery room (p<0.05)	
Fabian et al.^17^ 2005 Sweden Cohort	Overall birth experience (‘very positive’ and ‘positive’ versus ‘both positive and negative’ and ‘negative’ and ‘very negative’)		Women in the control group were more likely to have a less positive birth experience (p=0.08)
López-Gimeno et al.^24^ 2022 Spain RCT	MCSRS^f^		The mean global satisfaction was high in the control group and similar to that in the intervention group (p=0.224)
**Knowledge and misunderstandings**			
Munro et al.^11^ 2018 Canada RCT	Ten knowledge statements about outcomes related to epidural analgesia, each scored as ‘True’, ‘False’, or ‘Don’t Know’.		Changes in scores were significant for both the intervention group (95% CI: 1.33–3.51) and the control group (95% CI: 0.25–2.28)
Cherel et al.^12^ 2022 France Cohort	The principle of discordance	Knowledge was much improved in the intervention group (p<0.001)	
Shishido et al.^15^ 2020 Japan Non-RCT	Knowledge of labor analgesia was assessed by asking the women true/false questions.	The mean score of knowledge of epidural anesthesia was significantly higher in women assigned to the intervention group than in women assigned to the control group (p<0.001)	
Stewart et al.^18^ 2003 England RCT	Their knowledge was scored as none, moderate, or good, using a standardized scoring system, in which each item had several specific points of information on which the women were questioned	This only reached statistical significance for epidural top-ups for emergency cesarean section in the intervention group	Women in the intervention group were more knowledgeable about all the techniques, except for pethidine
Swan et al.^19^ 1994 Australia Cohort	They were asked initially if they recalled the anesthetist having no discussion and what risks they could recall	The intervention group had significantly better recall of each specific risk (p<0.05)	75% of women in the intervention group recalled a discussion concerning epidural risks compared with 62.5% in the control group (p>0.05)
Raynes-Greenow et al.^21^ 2010 Australia RCT	Knowledge of labor analgesia was assessed by asking the women true/false questions	A significant difference in knowledge scores was observed between the intervention and control groups (95% CI: 3.7–13.4)	
Raynes-Greenow et al.^22^ 2009 Australia RCT	Using 20-item true/false questions		Women’s knowledge scores improved significantly by between 10 and 13 points between baseline and primary outcome measures; however, no differences in the average change in scores were observed between the groups (95% CI: 5.9–0.34)
Togioka et al.^23^ 2019 USA RCT	The mean change between the 12-item total pre- and post-randomization survey scores regarding misconceptions associated with labor epidural analgesia	The intervention group had significantly lower rates than the control group in both Hispanic (p<0.001) and non-Hispanic (p=0.005) cohorts	
**Anxiety and fear**			
Maimburg et al.^13^ 2010 Denmark RCT	DFS ^h^		No differences were found in the two groups’ single-item scores or sum scores on the DFS
Raynes-Greenow et al.^21^ 2010 Australia RCT	Short Spilberger anxiety scale		No significant differences were observed between the anxiety scores of the two groups (95% CI: 2.15–1.5)
Raynes-Greenow et al.^22^ 2009 Australia RCT	The six-item short form of the state scale of the Spielberger State-Trait Anxiety Inventory		Anxiety scores for women in both groups remained similar to the pre-intervention scores and did not differ between the groups (95% CI: 2.23–1.74)
**Conflict**			
Shishido et al.^15^ 2020 Japan Non-RCT	DCS ^a^	Women assigned to the intervention group had a significantly lower mean DCS score than women assigned to the control group (p<0.001)	
Raynes-Greenow et al.^21^ 2010 Australia	DCS ^a^		Both groups decreased; however, no difference was observed between the groups (95% CI: 3.07–1.07)
Raynes-Greenow et al.^22^ 2009 Australia RCT	DCS ^a^		Compared with the baseline data, the mean Decisional Conflict Scores, post-intervention, were significantly reduced in both arms of the trial (p<0.0001), although this reduction in decisional conflict was not different between the two groups (p=0.376)
**Degree of participation in the decision-making process**			
Raynes-Greenow et al.^21^ 2010 Australia RCT	Control preference scale To collect on monitoring adherence to the intervention, i.e. had the women used the decision aid (read all, some, or none of it), listened to the CD, and completed the worksheet	There were significant differences between whether or not they had considered their care providers’ opinions when making their labor analgesia decisions (95% CI: 0.64–0.95)	Overall, 98% of women regardless of the trial group wanted to be actively involved in their labor analgesia decision-making. Most women in both groups planned to make their decisions by themselves, without necessarily consulting their care providers
Raynes-Greenow et al.^22^ 2009 Australia RCT	Women were asked whether they had used ‘all’ of the materials they received, ‘most’, ‘some’, or ‘hardly any’. Compliance was defined as high when women had read and used all the interventions they received, either the booklet and worksheet or the booklet, worksheet, and CD.		Both groups reported high compliance with using the intervention, and no significant differences were observed between the groups (p=0.369)
**Feeling supported by others, control of emotions and attitudes**			
Shahveisi et al.^14^ 2023 Iran RCT	SCIB^i^	The mean scores of the subscales of internal control, external control, and professional support in the intervention group were significantly higher than those in the control group, postintervention (p<0.001).	
**Planned to or decided to use epidural analgesia during delivery**			
Munro et al.^11^ 2018 Canada RCT	A single-item question: ‘For your next birth, would you consider an epidural?’ scored as ‘Yes’, ‘No’, and ‘Unsure’		The preference for epidural analgesia for the next birth did not change overall after reading the pamphlet or reading either the short or the detailed version
Maimburg et al.^13^ 2010 Denmark RCT	Use of epidural analgesia	Women in the intervention group used epidural analgesia less than those in the control group (p<0.01)	
Shishido et al.^15^ 2020 Japan Non-RCT	The decision on using anesthesia for labor during vaginal delivery (i.e. with epidural anesthesia, without epidural anesthesia, or undecided) was determined using a single question		In the intervention group, the proportion of women with epidural anesthesia increased from 40.3% to 54.0%, and the proportion of women without epidural anesthesia rose from 29.5% to 39.9%. In the control group, the proportion of women with and without epidural anesthesia slightly increased from 26.7% to 27.5% and from 32.6% to 33.6%, respectively
Fabian et al.^17^ 2005 Sweden Cohort	Use of pain relief techniques	The difference in the lower rate of epidural anesthesia in the control group was statistically significant (p=0.03)	Women in the intervention group generally used more pain relief techniques during labor, pharmacological as well as non-pharmacological, compared with those in the control group
Brixval et al.^20^ 2016 Denmark RCT	Use of pain relief techniques		Among the women in the intervention group, 30.5% received epidural analgesia compared with 29.1% in the control group (p=0.41)
Raynes-Greenow et al.^21^ 2010 Australia RCT	Intentions or plans to use analgesia were measured using a five-point nominal scale from ‘no plans to use’ to ‘very definite plans to use’. The actual use of analgesia was measured using self-report. The proportion of women who reported ‘no plans to use’ or ‘not going to use’ an analgesic option was compared to their actual use		No significant differences were observed in the analgesic use between the groups. No significant differences in analgesia intentions and use were observed between the groups
Togioka et al.^23^ 2019 USA RCT	Use of epidural labor analgesia	In the Hispanic cohort, the intervention group was significantly more likely to receive epidural analgesia compared with the control group (p=0.029)	In the non-Hispanic cohort, no difference in epidural analgesia use was observed between the intervention and control groups (p=0.616), but epidural analgesia use was significantly more prevalent in the non-Hispanic cohort (41 of 50 intervention vs 42 of 49 control)
López-Gimeno et al.^24^ 2022 Spain RCT	Methods of pain relief	A lower proportion of women in the intervention group used pharmacological methods than those in the control group (p=0.001), and a greater proportion of women was observed in the intervention group combined pharmacological and non-pharmacological methods (p=0.001)	
López-Gimeno et al.^25^ 2024 Spain RCT	Participant preferences	The intervention group was significantly more likely to prefer epidural analgesia compared with the control group (p=0.023)	

a Decisional conflict scale.

b Pregnancy and maternity care patients’ experiences questionnaire.

c Preterm birth experience and satisfaction scale.

d Women’s views of birth labor satisfaction questionnaire.

e Satisfaction with the decision.

f Mackey childbirth satisfaction rating scale.

g The labor agentry scale.

h The delivery fear scale.

i Support and control in birth.

*Satisfaction with the birth experience*

Six studies^12-14,16,17,24^ measured satisfaction with the birth experience, and the interventions implemented were a combination of presentation and discussion^12^, a combination of group education and discussion^13^, a combination of counseling and lecture^14^, a combination of leaflet and video^16^, group education^17^, and a combination of leaflet and counseling^24^. Of these, three studies^12,14,16^ revealed a significant difference in effectiveness, and the interventions implemented in studies where an effect was found were a combination of presentation and discussion by nurses and anesthesiologists^12^, a combination of counseling and lecture by midwives^14^, or a combination of leaflet incorporating SDM methods and video^16^. No studies have reported results on group education increasing satisfaction with birth experience^13,17^. Two studies implemented interventions based on SDM methods, and both results demonstrated that the intervention group was significantly more effective than the control group^14,16^ (Table 2).

*Knowledge and misunderstandings*

Eight studies^11,12,15,18,19,21-23^ measured knowledge and misconception changes due to decision aids, and the interventions implemented were leaflets only^11,15,16^, a combination of presentations and discussion^12^, group education^19^, a combination of leaflets and audio guides^21,22^, and a combination of videos, leaflets, and counseling^23^. Six^12,15,18,19,21,23^ of these studies revealed significant differences in effectiveness. All of the methods were effective; however, some studies that used leaflets alone or a combination of leaflets and audio instructions demonstrated no significant differences. In the study that reported no significant difference^11,22^, only leaflets were distributed to the control group, and knowledge scores increased in both groups^11^. As for satisfaction, knowledge scores tended to increase even with media-only interventions^15,18,21^ (Table 2).

*Anxiety and fear*

Regarding anxiety and fear of childbirth, three studies used it^13,21,22^: two used a combination of a booklet and an audio guide^21,22^, and the remaining one combined lecture and discussion^13^. None of the three studies found a significant reduction in anxiety or fear, and no significant difference was observed between the two groups (Table 2).

*Conflict*

Three studies^15,21,22^ measured changes in conflict, and the intervention implemented was either a leaflet only or a combination of a leaflet and an audio guide. Of these, one study revealed a significant difference in effectiveness, and the intervention implemented in the effective study was a leaflet only^15^. In both studies^21,22^ reporting no significant difference, only leaflets were distributed to the control group, and conflict levels decreased in both groups. As for satisfaction, knowledge, and misunderstandings, conflict levels tended to decrease even with an intervention that used only media (Table 2).

*Level of involvement in the decision-making process*

Two studies^21,22^ measured participation in the decision-making process, and both interventions combined a leaflet and an audio guide: one showed a significant difference and one reported that the intervention group was significantly more likely to report making the decision themselves after seriously considering their care providers’ opinion compared to the control group who reported that they had made their labor analgesia decision by themselves^21^. This indicates that the decision-making support had been implemented effectively. In the study that revealed no significant difference, the control group was also given only leaflets, and the conflict level decreased in both groups^22^. Media-only interventions tended to increase participation in the decision-making process^21^ (Table 2).

*Feeling supported by others, control of emotions and attitudes*

One study measured the perceived support from others and emotional and attitudinal regulation, which combined counseling and verbal explanations and found a significant difference in effectiveness. Moreover, the intervention in this study was based on SDM methods^14^ (Table 2).

*Planned to or decided to use epidural analgesia during delivery*

Nine studies^11,13,15,17,20,21,23-25^ analyzed whether epidural analgesia was planned to be used or used during labor, and the interventions used were a leaflet only^11,15^; a combination of group education and discussion^13^; group education^17^; a combination of leaflet and audio guide^21^; discussion^20^; a combination of video, leaflet, and counseling^23^; and a combination of leaflet and counseling^24,25^. Two studies reported that the planned or actual use of epidural analgesia was significantly lower in the intervention group, and the interventions used were a combination of group education and discussion^13^ or a combination of leaflet and counseling^24^. Conversely, three studies demonstrated that the planned or actual use of epidural analgesia was significantly higher in the intervention group, and the interventions used were group education^17^; a combination of video, leaflet, and counseling^23^; or a combination of counseling and leaflet^25^. The combination of counseling and leaflets increased the number of women who combined epidural analgesia with non-pharmacological methods such as breathing techniques^25^. Of the three studies reporting that the planned or actual use of epidural analgesia was significantly higher, one was a US study with a Hispanic cohort. The study background was that Hispanics were already using epidural anesthesia at a low rate due to factors such as medical disparities^23^. In another case that increasingly used anesthesia, the intervention reduced the use of epidural anesthesia; however, the use of anesthesia in the control group was low, to begin with. Therefore, the intervention group used more anesthesia than the control group^17^. In the remaining case with high anesthesia use, the intervention decreased the desire to use anesthesia in both groups; however, more people wanted it in the intervention group than the control group at baseline^25^ (Table 2).

## DISCUSSION

### Literature summary

This scoping review determined what is known about decision aids and their effects on women choosing epidural analgesia during delivery. A total of 15 articles, including randomized controlled trials, non-randomized controlled trials, and cohort studies, were included for analysis based on the eligibility criteria.

Decision aid methods included leaflets, counseling, lectures, presentations, discussions, videos, group education, and audio guides. These were mainly delivered during the second or third trimester of pregnancy. Most were delivered by nurses; however, some were delivered by doctors or health professional teams and by media-only. When interventions were divided into those providing media such as leaflets and those involving expert interaction such as discussion or counseling, media-only interventions increased satisfaction, knowledge (and reduced misconception levels), participation in the decision-making process, and reduced conflict levels, whereas adding expert interaction further increased satisfaction.

### Effectiveness of decision support

Regarding the intervention timing, most articles included were in the middle or late stages of pregnancy, rather than in the early stages. Previous studies have reported that late pregnancy was considered the ideal time to be given information about labor analgesia^26^. Furthermore, 73% of women stated that they would want counseling on analgesic options from their obstetric providers during the second or third trimester^27^. Furthermore, of the studies included in this review, 66% of women wanted information about pain relief during labor and the opportunity to discuss the information given in the late stages of pregnancy^15^. Given the above studies, decision support interventions may be better provided in the middle to late stages of pregnancy^18^, when women can particularly consider labor as their concern, rather than in the early stages of pregnancy. Regarding the implementers of decision support, many interventions in this study were provided by nursing professionals, such as midwives and nurses; however, a significant difference was detected between anesthesiologists alone and nursing professionals alone, with some interventions being effective and others not, and the results were mixed. In addition, results of the media-only intervention and the intervention involving midwives did not provide indicators of conflict, participation in the decision-making process, a sense of support from others, or control of emotions and attitudes, and comparisons between professionals were not possible; therefore, concluding which professional provides better decision-making support is challenging.

The literature involved in this study reveals that satisfaction or knowledge scores did not decrease or conflict levels increase in items such as information satisfaction, knowledge and misunderstandings, and conflict, when media such as pamphlets were used alone. Consequently, depending on the content and purpose of the media provided, intervention by medical professionals is not essential and that effects can be obtained with media alone.

The results showing a tendency for knowledge to improve by intervention, as well as the results of an increase in the sense of support from others and the sense of control over emotions and attitudes, suggest that in case of available information, it is easier to feel supported during labor and the possibility of using the information obtained on one’s own. Additionally, for the planned or actual use of epidurals, interventions may reduce the desire to use or use them, and increased knowledge on the risks and side effects of epidurals may increase the possibility of avoiding the use of epidural anesthesia. Conversely, the result that the intervention group used anesthesia more significantly than the control group in terms of planned or actual use of epidural anesthesia was because Hispanics use anesthesia less frequently during delivery than Whites. The purpose of the intervention may have differed depending on the original use of epidural anesthesia during delivery in the field and the target population. That may have resulted in different results. Although the intervention is the same, the expected effects may differ depending on the study background. In fear and anxiety, no method revealed a clear difference. Even if the patient temporarily calms down by asking questions, gaining knowledge, or talking, anxiety is likely to be remembered again as time passes or time approaches, and may be difficult to resolve with the intervention.

In recent years, SDM^28^, which refers to the process in which clinicians and patients work together to clarify the treatment, the management, or self-management support goals and share information about options and preferred outcomes to reach mutual agreement on the best course of action, has also attracted attention. All literature that clearly defined methods based on SDM in this study showed significant differences in satisfaction with the birth experience and feeling supported by others, control of emotions and attitudes; however, the number of studies is small. Therefore, the effects cannot be clarified. In addition, although research on SDM has focused mostly on the creation of tools to support patient involvement in decisions and much less on how to create a culture where professionals espouse SDM as a skill and routinely use these tools^29^, the universal methodology cannot be clarified. Therefore, research methods vary depending on the literature, and the effects may differ, warranting further research.

### Limitations

This review has limitations in generalizing the results of simple comparisons because the scales used to measure effectiveness were non-uniform. In addition, for some outcome items the literature articles are few, and the results have not been fully examined. Regarding the intervention timing, many studies have been conducted in the mid to late stage of pregnancy, indicating that intervention during this period is effective. However, no studies have compared the intervention timing; thus, further research is needed. This study did not evaluate the risk of bias; therefore, the results of the included literature may have been over- or under-evaluated. The present review only referred to studies on epidural anesthesia and excluded other pharmacological pain relief methods.

## CONCLUSIONS

According to this scoping review, decision support is provided through leaflets, counseling, discussion, and various other educational methods, with leaflets being the most commonly used approach. Furthermore, it is often implemented in the later stages of pregnancy and is generally implemented by nurses. However, this review did not compare the intervention timing; therefore, comparing the effects by timing is essential in the future. Furthermore, commonly investigated effects include the use of epidural anesthesia, knowledge and misunderstandings, and satisfaction. Regarding effective methods, none of the methods presented here reduced satisfaction or knowledge, and all of them may be effective. The effectiveness of the method may vary from person to person; however, further study is warranted regarding which methods are effective for different people.

## Data Availability

Data sharing is not applicable to this article as no new data were created.
